# A Bayesian spatio-temporal model of variation in homicide rates for El Salvador

**DOI:** 10.1371/journal.pone.0330215

**Published:** 2025-09-24

**Authors:** Carlos Carcach

**Affiliations:** The Center for Public Policy, Escuela Superior de Economía y Negocios, Academic Building, Calle Nueva a Comasagua, Santa Tecla, El Salvador; Imperial College London, UNITED KINGDOM OF GREAT BRITAIN AND NORTHERN IRELAND

## Abstract

Most violence in El Salvador has been attributed to gangs. After being one of the most violent societies worldwide, in 2023, the country’s homicide rate reached a low 2.4 per 100,000 population. The decline is the outcome of a security plan by government that has attacked gangs directly under a state of exception. Nearly 80,000 gang members have been incarcerated. For the past 30 years or so, Salvadoran authorities and politicians alike, have participated in negotiations with gangs to reduce violence and gain electoral support in exchange for benefits for their members. This research studies violence as the outcome from the activities by gangs, politicians and governments, their interactions, and communities intervening in the realization of these interactions. As most data required for explaining these processes is either inexistent or difficult to access, a hierarchical Bayesian model was implemented for the spatio-temporal evolution of homicide with random effects that account for omitted variables at the level of local areas and time periods. The results support the view that unobserved covariates related to the district patterns of homicide have evolved over time. Two cycles appear in the evolution of homicide over the period under study, one from 2003 through to 2012, and another starting in 2013 and still going on at the time of writing. This finding reinforces the view that timing of government-gang negotiations drove the behavior of homicide rates in El Salvador during 2002–2021 together with the growth and expansion of gangs as seen from clustering of high-risk districts over time. The incarceration of scores of members and collaborators has both incapacitated gangs as key producers of violence, and deterred other forms of crimes. As a next step, the government should build collective efficacy, in particular among disadvantaged communities, to restrain the formation of gang-like groups in the time to come.

## Introduction

A total 111,166 homicides occurred in the Central American country of El Salvador over the twenty-five years elapsed between 1994 and 2018 [1], equivalent to an overall rate of 73.6 homicides per 100,000 population. The situation changed over the five-year period from 2019 to 2023 when the country recorded a total of 5,474 homicides and the overall rate declined to 17.3 per 100,000 population. During the latter period, the annual homicide rate came down from 38.2 in 2019 to 2.4 per 100,000 in 2023. This decrease has taken place within the context of the current government´s “Plan Control Territorial” (PCT) being enforced through a state of exception decreed in March 2022, and still in place at the time of writing.

Historically, most of El Salvador’s violence has been attributed to the settlement, growth and territorial expansion of gangs, mainly the “Mara Salvatrucha” (MS-13) and the two factions of the “Barrio 18” (18^th^ Street) gang. Before 2019, gangs were believed to be responsible for at least 50 per cent of murders and to have 60,000 active members plus nearly 400,000 collaborators and close relatives [2]. This represented between 1 and 7 per cent of the total population.

Since around 1998, the different Salvadoran governments have struggled to bring the homicide rate down by trying different approaches to control gangs. From 1998 through to mid-2009, when government was held by the rightwing ARENA political party, the rate was 52.7 homicides per 100,000 population. In 2003, the Salvadoran government implemented the so-called “Mano Dura” (Iron Fist) policy, which intensified law enforcement operations against gang members. Under this policy, gang membership was criminalized, and law enforcement officers were granted increased authority to arrest individuals based on suspicion of gang involvement or gang-related tattoos. The policy led to mass arrests and increased penalties for gang-related crimes [3]. Different forms of the Iron-Fist anti-gang policy were in place until 2009.

From mid-2009 through to mid-2019, when the leftwing FMLN party was in government, the average homicide rate was 58.4 per 100,000. Some noticeable variations occurred within this 10-year period, the first being a government backed truce taking place between 2012 and mid-2013, period during which the homicide rate dropped down to 37.1 per 100,000. The second was an increase in the homicide rate between mid- 2013 and August 2015 to 69.1 per 100,000 following the government back off from the truce and the adoption of a strategy based on a frontal combat to gangs.

In August 2015, the Constitutional Tribunal of the Salvadoran Supreme Court of Justice declared the MS-13 and “Barrio-18” gangs terrorists. Such a resolution forced Salvadoran judges to apply the Law Against Acts of Terrorism to members of these groups and also to their apologists and financers [4].

By 2015, gangs had presence in nearly 90 per cent of districts – formerly municipalities. As expected, extortion to civilians, public transport and local businesses was the economic engine behind gangs. In 2015, one out of four Salvadorans reported that they had been the victim of extortion. Gang members would often go as far as to execute individuals or their friends and family when their payment was overdue or insufficient. At least 80 per cent of small businesses claimed that they paid extortion fees to the gangs, forcing some to close or go bankrupt. Extortion by gangs was identified as a major driver for the forced disappearance and the illegal migration to the United States of America of many Salvadorans [5–7]. Peñate-Guerra et al (2016) put at nearly $US 4 billion the annual cost of extortion to the Salvadoran economy [8].

The way gangs are organized allows for incarcerated leaders to command “clicas” or “canchas” on the streets on how to collect and distribute extortion funds and indicate them who should be killed for failure to meet payments, or any other reason. Often, gangs within prisons sparked violence in the streets and controlled violence in communities across El Salvador.

Concurrently with their efforts to regulate homicides and other crime by tackling gangs, for the past 20 years or so, Salvadoran authorities have also facilitated secret dialogues and negotiations with imprisoned gang leaders to reduce violence and gain electoral support in exchange for benefits for those leaders [9]. One of such negotiations, the so called “truce”, occurred in 2012 and brought a decline in the annual homicide rate from 71.2 in 2011 to 40.6 in 2013. The “truce” was the event that enabled gangs to become political actors. When the government backed off the truce in mid-2014 and declared a war against gangs, the homicide rate increased to 63.1 in 2014 and reached a peak of 106.3 per 100,000 in 2015. Gangs learned about using violence as the bargaining chip in their negotiations with authorities and politicians.

In March 2016, members of the “Barrio-18” gang murdered 11 workers of an electrical company and a farmer in a rural area located thirty minutes from the capital city, San Salvador [10]. The government used this event to argue for the need to confront an exceptional threat with the exceptional measures that were approved in April 2016. The so-called “extraordinary security measures” included reforms to criminal legislation aimed at facilitating the conviction of gang members and their collaborators; the creation and deployment of special combined police-army forces to neutralize gang actions and capture gang leaders; and application of special, transitory and extraordinary provisions in some of the country´s prisons to break the communication mechanisms by which the imprisoned gang leaders directed and coordinated extortions, murders, and other crimes on the outside.

The government attributed the fast decline in the numbers of homicides from an average twenty-per-day in March 2016 to an average eleven-per- day in April 2016 to the “extraordinary measures”. To others, the decline was due to a temporary cease-fire announced by the MS-13, the 18^th^ Street “Sureños” and the 18^th^ Street “Revolucionarios” just before the new legislation was enacted together with the warning by gang representatives that they would react with greater violence if the government moved forward with the “extraordinary measures”. However, the government reiterated its unwillingness to enter in negotiations with the gangs [11].

In September 2020, a journalistic investigation revealed that negotiations between officials of the newly elected Bukele government and the MS-13 had been active since 2019 [12]. The approval of the current state of exception followed a crime spike in March 25–27, 2020 when gangs allegedly murdered 87 people. Two other gang-perpetrated known massacres took place in April 2020 and November 2021 involving 76 killings over four days and 45 people murders in three days, respectively [13].

The state of exception has been instrumental to implement a security plan to attack gangs directly. It restricts some constitutional guarantees and has resulted in over 80,000 alleged gang members being arrested. However, the decline in homicide started soon after 2016 when the previous FMLN government introduced the already referred “extraordinary measures”. It seems reasonable to say that the PCT has been a more extreme and efficacious version of its predecessor, and that it is a continuation of the war-on-gangs policy initiated by the previous FMLN government.

Despite the indisputable drop in homicide during 2019−2023, its underlying reasons are not cut clear. A number of questions remain unanswered. First, if homicide seemed to be highly correlated with the unobserved numbers of gang members, and gangs had territorial presence in nearly 90% of the 262 country districts, why these groups did not retaliate in response to the incarceration of scores of its alleged members. Is it reasonable, as proposed in [14], to attribute this to the speed and scale of the crackdown by the army and the police that took the MS13 and “Barrio-18” by surprise and the gangs failed to mount a coordinated response? And as a consequence, that the numbers of gang perpetrated homicides drop because a sizable portion of gang members ended up in jail, were forced into hiding, or fled the country? Second, if gangs have accounted for nearly half the total homicides in El Salvador during the last 30 years or so, what about the remaining violent deaths? Did these murders dropped to nearly zero due to some sort of generalized deterrence effect associated with the state of exception, or some of them are not being included as part of the official counts of homicide? Third, given the history of negotiations between governments and these groups, would it be possible for the massive incarceration of alleged gang members, together with the drop of homicide, to be part of a more complex agreement between the current Salvadoran government and the gang leaders, either incarcerated or at large?

### On this research

Our main hypothesis is that homicide evolution has been driven mainly by gang related violence and their interactions with politicians and governments as well. There is evidence that in the United States of America (U.S.), gang homicides have strong overall effects on the rates of lethal violence beyond those due to specific structural characteristics linked to social disorganization, relative socioeconomic disadvantage, and neighborhood environmental conditions [15]. In Latin America and the Caribbean, homicides related to organized crime and gangs have been found to be more volatile than other homicides, and high homicide rates are also usually associated with a proportionately higher number of homicides related to organized crime and gangs. Where there is a higher density of criminal organizations and gangs, there is a higher risk of homicidal violence [1]. Studies for the U.S. show gang homicides as exhibiting greater spatial concentration and use of firearms when compared with homicides in general [16–17]. In El Salvador, firearm related homicides and gang related homicides accounted for 78.6% and 36.7% of the total homicides recorded between 2014 and 2021, respectively [18].

This research is to study the evolution of homicide in El Salvador within a hierarchical Bayesian spatio-temporal framework over the 2002–2021 period. A Bayesian approach allows one to account formally for uncertainty at each of the data, process and parameter levels, where these levels are linked in a probabilistic consistent fashion [19]. We pay attention to localized spatial-temporal patterns by incorporating spatio-temporal interactions. In particular, we deal with the case when district-level deviations from the global trends are assumed to be correlated with their neighbors, both in space and time. We model hidden factors whose effects go beyond the geographical border of one or more districts and also persist for more than one period of time. Such an approach is the most efficient for extracting information from data, especially in the case of rare-events or less populated regions, since the estimation of the homicide risk for a district-period is performed not only based on the locally observed data but also on that for neighboring districts and time periods [20]. A combined space-time approach to account for additional variation in homicide is amenable to the idea of violence as an outcome from the activities by gangs, politicians and governments, the interactions they establish, and that their unobserved states together with the unobserved community composition can intervene in the realizations of these interactions. Our research updates and extends the findings in [21].

Several studies on the temporal and spatial distribution of homicide or other forms of violence in Latin America have been published in the specialized literature, especially for Brazil [22–25], Colombia [26,27]; Ecuador [27,28], El Salvador [21,29–32], and Mexico [33–35]. All these studies have used aggregated data for municipalities, districts or other kinds of administrative areas. With the exception of [27,21,30–32], none of these studies used Bayesian random-effect based methods to either model dependence and heterogeneity at the area or time levels, or to consider area-time interactions while accounting for unobserved variables.

## Materials and methods

### Data

The data for this study consisted of the numbers of homicides occurring at each of the 262 districts or former municipalities across El Salvador over the twenty-year period spanning from 2002 to 2021, and the numbers of allegedly gang members detained by district over the eight-year period from 2011 to 2018. Data on homicide was obtained from the *Policía Nacional Civil* (*PNC*) for the periods running from 2002 through to 2007 and from 2014 through to 2021, and the *Instituto de Medicina Legal* (*IML*) for the remaining years (S1 File: Homicide_Rates_per_10,000, 2002–2021). The *PNC* was the source for data on numbers of gang detentions (S2 File:Detentions_Alleged_Gang_Members_2011–2018). Official district population projections obtained from the *Oficina Nacional de Estadística y Censos* (ONEC)- formerly *Dirección General de Estadística y Censos* (S3 File: Official_population_projections_2002–2022) – were used in the computation of crude and expected homicide rates (S4 File: Observed and expected homicides, 2002–2021). The municipal maps included in this paper were based on the El Salvador official municipal limits obtained from the shapefile and its associated database format file and machine-readable version file that are publicly available from the *Centro Nacional de Registros* (CNR). The author processed the shapefile to develop the maps included in the paper (S8a File:_El Salvador municipal map-dbf (XLSX), S8b File:_El Salvador municipal map-shp (XLSX), S8c File: El Salvador municipal map-shx (XLSX)). CNR published geographic data are provided at no cost to use for non-commercial purposes [36].

### Exploratory analysis

**Fig 1:**
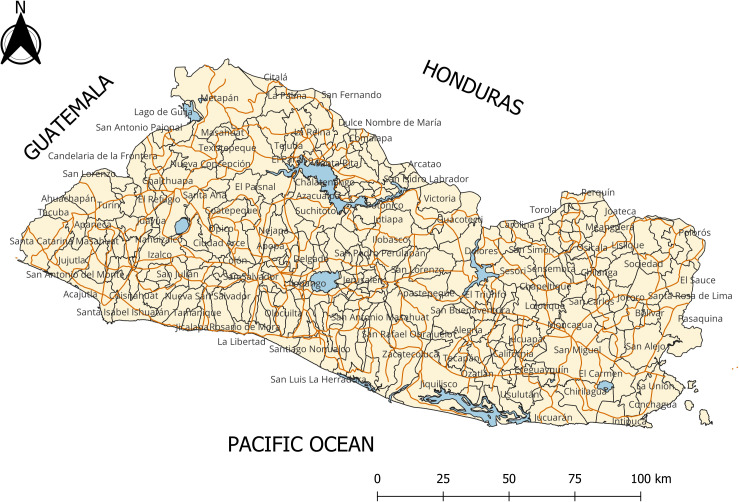
Map of El Salvador. Data source: S8b File:_El Salvador municipal map-shp (XLSX).

With a total population of about 6.5 Million distributed over 21,041 Square Kilometers; El Salvador is organized in 262 districts, formerly municipalities, varying widely in terms of size and number of residents. El Salvador extends on the Pacific coast sharing borders with Guatemala on the West, and Honduras on the North and the East. Roughly, its territory divides into 3 well differentiated regions: A coastal region running along the Pacific Ocean; the most densely populated central plateau, covering 85 percent of the territory; and the northerly mountain ranges. These regions define climatic and environmental zones characterized by varying land uses, economic activities and potentials. Fig 1 shows the map for El Salvador with the boundaries and names for its 262 districts and the main roads crossing the country. Qgis 3.42.1 was used to develop this map.

**Fig 2:**
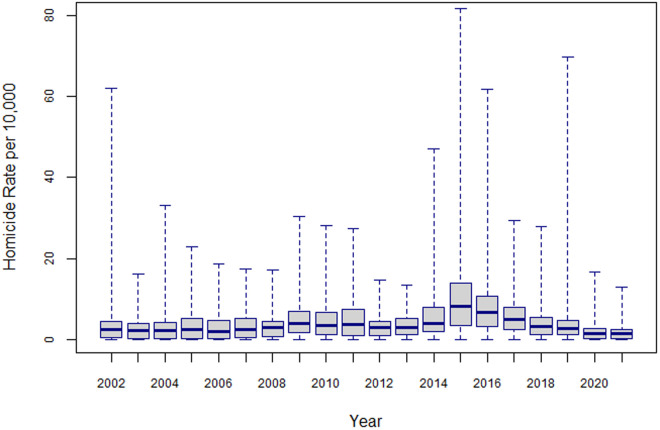
El Salvador, Crude Homicide Rates per 10,000 population (2002-2021). Data source: S1 File:_Homicide_Rates per 10,000,_2002-2021.

The boxplot in Fig 2 shows the temporal trend in the crude homicide rates between 2002 and 2021.

The data in Fig 2 and Table 1 highlight several features of the homicide rates over the 2002–2021 period. First, the homicide rate per 10,000 increased from 4.3 in 2002 to 7.0 in 2011 varying around an average value of 5.7 per 10,000. Following a decline to 3.7 in 2012–2013, the rate recovered its upward trend during 2014–2016 when it varied around an average 8.3 per 10,000. Finally, from 2017 and until 2021, the homicide rate decreased from 6.1 to 1.7 homicides per 10,000. Second, there were three years over the 2002–2021 period when the homicide rate reached relatively low values (3.9 in 2003, 3.6 in 2012 and 4.0 in 2013). The facts that 2003 was the year when the Salvadoran government implemented its Iron-Fist policy, and that 2012–2013 was the period during which the “truce” was in place seem to relate to such low rates. Third, there seems to be a decreasing trend in the between-district variability for the annual homicide rates for specific periods – between 2004 and 2008, between 2009 and 2013, between 2015 and 2018, and since 2019. These trends in variability suggest that homicide rates may be affected by heterogeneity, both across districts and over time. Finally, the proportion of districts with no homicides shows a downward trend between 2002 and 2015, and an upward trend between 2016 and 2021.

**Table 1:** El Salvador, crude homicide rates per 10,000 population (2002-2021). Data source: S1 File_Homicide_Rates per 10,000,_2002-2021.

	Overall Rate per 10,000 Population	Median	Standard Deviation	Interquartile Range	Minimum	Maximum	Districts with no homicides (%)
2002	4.28	2.84	2.43	2.76	0.00	14.52	25.2
2003	3.92	2.90	2.55	2.89	0.00	15.82	30.2
2004	4.95	3.09	3.65	3.33	0.00	33.07	27.1
2005	6.57	3.67	4.33	5.27	0.00	22.83	28.2
2006	5.13	3.25	3.23	3.64	0.00	18.37	28.6
2007	6.07	3.91	3.49	4.38	0.00	17.40	26.0
2008	5.20	3.57	2.91	2.87	0.00	17.23	24.0
2009	7.12	4.93	4.47	4.58	0.00	30.33	17.6
2010	6.47	4.63	4.13	4.86	0.00	28.01	20.6
2011	7.00	5.14	4.09	5.66	0.00	27.33	22.5
2012	3.59	3.56	2.20	2.54	0.00	14.51	22.1
2013	4.00	3.58	2.70	3.72	0.00	13.38	18.7
2014	6.21	4.72	5.59	6.41	0.00	47.07	11.8
2015	10.48	8.82	8.78	10.05	0.00	81.36	8.4
2016	8.26	6.92	7.91	6.98	0.00	61.74	9.5
2017	6.14	5.91	4.68	5.13	0.00	29.28	13.7
2018	3.95	3.95	3.38	3.68	0.00	27.64	21.4
2019	3.57	3.20	7.52	3.43	0.00	69.73	19.5
2020	1.95	2.13	2.29	2.17	0.00	16.38	30.2
2021	1.66	1.82	1.71	1.56	0.00	12.50	26.3

An analysis of the annual homicide rates using the *pacf()* and *Arima()*functions in the *forecast* R package [37–38], indicated that a second-order autoregressive model provided a satisfactory fit to the data.

**Fig 3:**
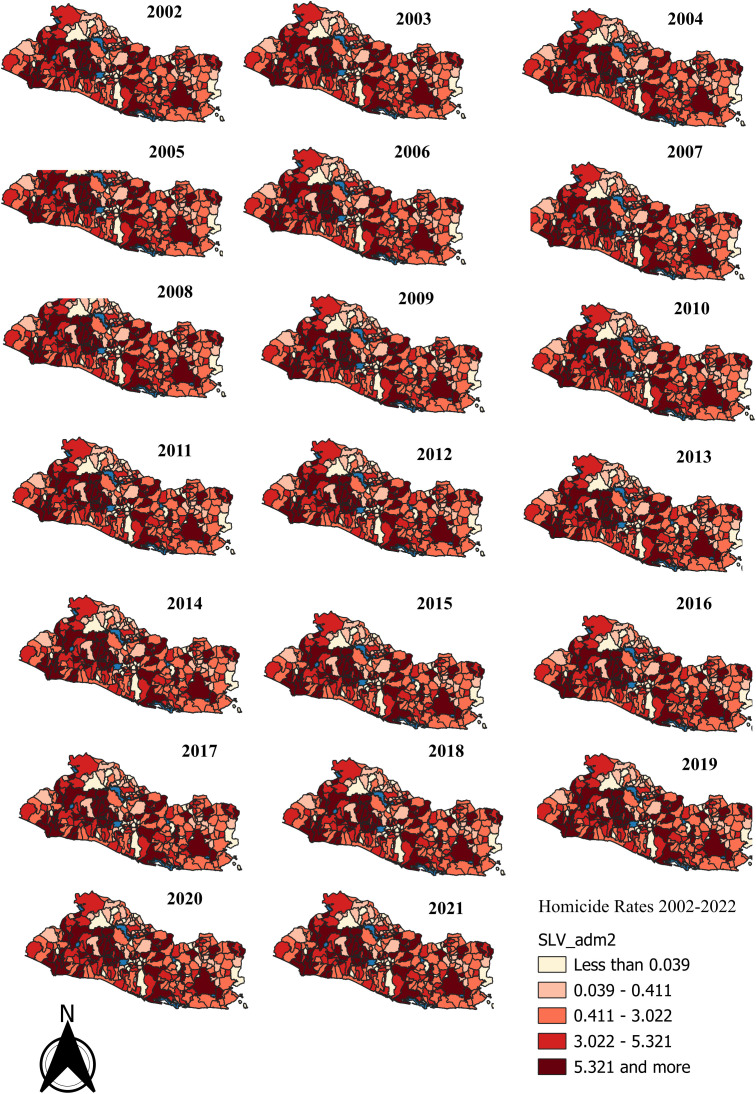
El Salvador, crude homicide rates per 10,000 population (2002-2021). Data source: S9a File _El Salvador homicide rates with geographic-dbf (XLSX), S9b File:_El Salvador homicide rates with geographic-shp (XLSX), S9c File:_El Salvador homicide rates with geographic-shx (XLSX).

Fig 3 shows maps of the crude homicide rates per 10,000 population over the 2002–2021 period. Qgis 3.42.1 was used to develop this map.

To test for the presence of spatial autocorrelation in the homicide rates, the Moran’s I statistic [39] was computed, and a permutation test was conducted. The permutation test had the null hypothesis of no spatial autocorrelation against the alternative of positive spatial autocorrelation. It was conducted using the *moran.mc()* function in the *spdep* R package [40]. The estimated Moran I statistic was 0.1841 and its associated *p-value* less than 0.01, indicating strong evidence of spatial autocorrelation between the district homicide rates.

The 262 districts are located over a total extension of about 21,000 square/kilometers or an average 80.7 square-kilometers per district. Population density is high. In 2021, the average district had 568.8 inhabitants per square-kilometer. Such a large concentration of people within relatively small territories suggests the possibility of intense social and economic interactions some of which may make some local areas to nurture violence engendering conditions related to gang activity.

**Fig 4:**
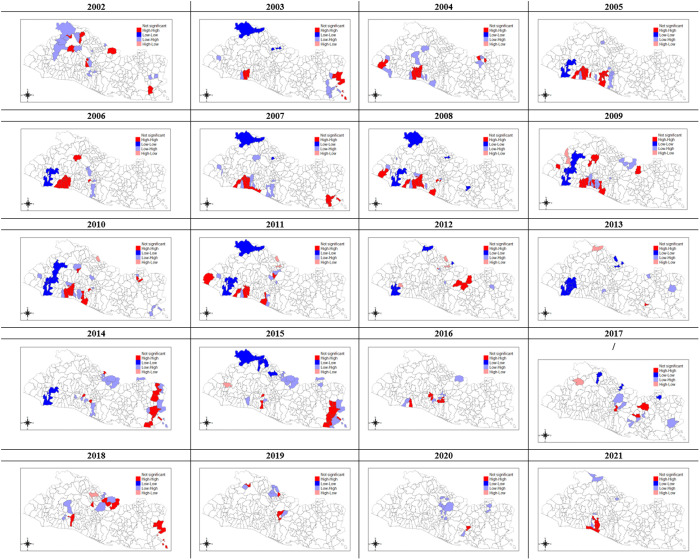
El Salvador, LISA p-values – Crude Homicide Rates per 10,000 Population (2002-2021). The legends in the maps correspond to the following values for the district-LISA-p-values: Non-significant (white), High-High (red), Low-Low (blue), Low-High (light-blue), and High-Low (light red).

Once gangs delimit, establish in and gain control of a territory they protect it from outside influence from the risks of murder by rival gangs and arrest by the police through checkpoint systems. Both the MS-13 and the Barrio 18 gangs impose restrictions on individual mobility to track down defectors and prevent the out-migration of residents from the controlled territory. Also, the checkpoints allow the gangs to extort, or impose taxes on, individuals and businesses that are allowed to enter or exit their territory. On the other hand, gangs claim to protect locals from other criminals and the police. Finally, in the absence of mobility restrictions and due to political convenience, central and local governments have often invested in infrastructure and social and educational programs in gang-controlled neighborhoods, not just in state-controlled areas. As shown in [41], gangs have a significant negative effect on socioeconomic development in the neighborhoods they control. Residents of gang-controlled neighborhoods have lower earnings and education attainment than those in non-gang areas; businesses located farther outside of gang neighborhoods have significantly higher profits than firms operating in the boundaries of gang areas; dropout rates in schools from a gang territory are larger than in schools outside a gang territory; also residents of gang neighborhoods are more likely to seek help from the gangs when they have a problem with public goods provision, a financial issue, or a security, civic, or legal dispute, and are more likely not to seek help from other instances, possibly out of fear that the gangs might punish them for complaining about their problems. This depletion of social capital is an important contributor to high levels of homicide by reducing a community’s ability to exercise informal and formal social control, and increasing levels of anomie and strain [42]. As a consequence, residents of neighborhoods in gang-controlled areas are expected to be less cohesive and less willing to intervene on behalf of the common good which reduces the neighborhood level of collective efficacy, which in turn is linked to increased violence. As the process of gang territorial expansion occurs over time and given the spatial correlation and space-time heterogeneity of homicide rates already noticed, there is a strong possibility for space-time interactions in the evolution of both gang activity and violence [43,44]. The presence of space-temporal interactions in homicide was visually assessed from *p-value* maps of the LISA statistic [45,46], by year. The districts with high homicide rates surrounded by high crime neighbors were shaded red on the maps in Fig 4. This map and all the other maps included in the paper were obtained using the *spplot()* function in the *sp* R package [47]. The maps indicate that the location of high crime clusters varied over time, as it was with the location of low crime clusters (blue shaded).

These patterns suggest the presence of spatial and temporal factors that are interdependent. This interdependence is central to such conceptual perspectives on violence as routine activities theory [48], and gang conflicts [49].

### Space-time model for homicide

Let the i index designate a district, (i=1,2,…,262), the t index, a specific year, (t=1,2,…,20), and let $Y_{it}$ denote the number of homicides recorded at district i in year t. $Y_{it}$ was assumed to arise from a Negative Binomial distribution with mean ${\mu_{it}=E}_{it}\theta_{it}$ and $\mu_{it}r$ variance, where r captures overdispersion in the data. The $\theta =\left(\theta_{1,1\;}{,\;\theta }_{1,2\;}\ldots ,\theta_{1,20\;},\;\ldots ,\theta_{262,20\;}\right)$ parameters represented unknown district-specific relative homicide risks, estimated with the (indirectly) standardized mortality rates (SMRs), and $E=\left(E_{1,1\;}{,\;E}_{1,2\;}\ldots ,E_{1,20\;},\;\ldots ,E_{262,20\;}\right),\;$the expected numbers of homicides at district i in year t. A same distribution was initially assumed at the first level of hierarchy, given by


$$Y_{it}|\;\theta_{it},\;r\;\sim NegBin\left(\mu_{it},r\mu_{it}\right)$$ (1)

The Negative Binomial is a convenient alternative to the Poisson distribution as it is suitable to accommodate the situation of highly variable homicide rates (refer to Fig 2) allowing for the estimates of relative risk to vary over districts and years. For rare events such as homicides, extra variation may arise either from nonconstant variance of the numbers of homicides, heterogeneity of risk levels between districts, and from the clustering of incidences in either space or time, or both. Heterogeneity and clustering may appear from the omission of variables that are relevant to explain variability of the homicide rates across districts and over time such as district characteristics with a potential to promote the settlement, growth and expansion of gangs, or periods when negotiations between governments and gangs have occurred. Data on variables related to gang presence, gang activity, or gang-government negotiations, are hardly to obtain or not available at all at the district level. There is a great number of both unobserved and unobservable variables peculiar to each district that induce variation in homicide risk across districts. Random effects help address the issues arising from omitted variables by accounting for variability within the data.

We allowed the district-year-specific risks to depend on random effects to accommodate spatial and temporal over-dispersion in the homicide counts [50,51]. These random effects allow the model to capture the impact of unobserved variables at the level of districts, time periods, or both.

At the second level, the logarithm of relative risk, $\theta_{\text{it}}$, was defined as


$$log\left(\theta_{it}\right)=\;\alpha +U_{i}+V_{i}+\;\gamma_{t}+\varphi_{t}+\delta_{it},$$ (2)

Where α is an intercept representing an overall relative risk following a fixed or flat default prior; $U_{i}$ is the unstructured or independent effect of district i, modeled as an independent and identically distributed normal variable; $V_{i}$ represents the spatially correlated effect of district i, following a conditionally autoregressive (CAR) distribution [52]; $\gamma_{t}\;$designates a temporally structured effect modeled as a second order random walk in time; $\varphi_{t}$ accounts for the year-specific unstructured effect, modeled with an independent and identically distributed normal variable; and $\delta_{it}\;$represents a non-separable space-time interaction.

Both, interpretation of the non-separable space-time interaction and assignment of a prior distribution to it, depend upon its specification. Four specifications are possible [53–55]:

- Type I where the interaction is between both unstructured district and time effects ($\delta_{it}=U_{i}\times \varphi_{t})$. Under this specification, missing covariates lack district-year structure, and unexpected high or low numbers of homicides in district i and year t are not associated with higher or lower numbers of incidents in other years, nor in neighboring districts. These are sudden or one-off events. $\delta_{it}$ is modeled as an independent and identically distributed normal variable.
- Type II corresponds to the interaction between the unstructured district and the structured year effects ($\delta_{it}=U_{i}\times \gamma_{t})$. Missing covariates are interpreted as having smoothly varying structure through time but do not vary over districts. Their effect on the outcome is temporary persistent but it is highly localized. $\delta_{it}$ is modeled as an independent and identically distributed normal variable.
- Type III refers to the combination of the structured district and the unstructured year effects ($\delta_{it}=V_{i}\times \varphi_{t})$. Missing covariates have smoothly varying structure across districts but their effects are restricted to the specific time period t. Under this definition, unexpected high or low numbers of homicides in district i and year t is associated with higher or lower counts in other neighboring districts but not in other years. The effects of missing variables are spatially structured but short-term. $\delta_{it}\;$follows an intrinsically conditional autoregressive (ICAR) distribution.
- Type IV interacts the structured district and the structured year effects ($\delta_{it}=V_{i}\times \gamma_{t})$. Missing covariates have smoothly varying structure across space, through time and space-time. Their effect on the outcome is spatially structured and temporary persistent.$\;\delta_{it}\;$was assigned an intrinsically conditional autoregressive (ICAR) distribution [56].

The $U_{i}$ random effect reflects the marginal dependence of the homicides occurring within a specific district. Similarly, the random effect $\varphi_{t}$ reflects the marginal dependence of the homicides taking place within a given year. Introduction of the spatial term, $V_{i}$, to capture location effects, induces spatial dependence among the homicides that occur within a specific district. The structured temporal term, $\gamma_{t}$, reflects a dependence among the murders over the years. Finally, inclusion of the space-time term, $\delta_{it}$, reflects the evolution of district patterns over time. The space-time interaction clustering is usually interpreted in terms of infectious processes

The third level of the hierarchical structure corresponds to the priors for the hyperparameters. We assigned the default R-INLA priors for the precision of the random effects, log(precisionssimlog−gamma(1,0.001) [57].

Models for the different specifications for the space-temporal term were fitted with the R-INLA package in R using the integrated nested Laplace approximation (INLA). INLA is a computationally less-intensive alternative to Markov Chain Monte Carlo (MCMC) methods to analyze spatially auto correlated data [58–60].

## Discussion

### Model fitting

Model fit was evaluated using the Deviance Information Criterion (DIC) [61] and the Widely Used Information Criterion (WAIC) [62]. The best fitting model should have both, the lowest DIC or WIAC, and a small effective number of parameters to estimate (pD or pW). Several models were examined to determine the best fitting model via DIC and WAIC, as seen in Table 2. The data indicate that there was a need for a model with a term for the space-time interaction, and that amongst those that included such a term, the space-time model with Type-IV interaction provided the best fit to the data as assessed from both its DIC and WAIC values.

**Table 2:** Model Comparison in Terms of Fit and Complexity.

Model	DIC	pD	WAIC	pW
No Space-Time Interaction	26,219.08	220.44	26,265.74	248.31
Type I – Space-Time Interaction	26,218.51	220.29	26,265.47	248.43
Type II – Space-Time Interaction	25,123.76	830.24	25,271.57	829.11
Type III – Space-Time Interaction	25,446.47	1,036.97	25,490.47	908.30
Type IV – Space-Time Interaction	24,875.07	839.43	25,029.58	848.54

Table 3 shows that the district-level relative risks of homicide varied around an average value of 0.553 (=exp(−0.591)) with a 95% credible interval [0.522,0.594]. The data in Table 3 indicate that as expected, 92.8% of the total variance of district homicide rates was due to the district-year interaction. As it was mentioned before, the model with Type-IV interaction is consistent with the notion of the missing covariates having an effect on the homicide rates that is structured across districts and persistent over the years. This kind of time variation in clustering effects was hypothesized to reflect the previously discussed patterns of settlement, growth and dispersion of gangs over time, and the effects of unobserved actions by governments, politicians and communities.

**Table 3:** Posterior means, standard errors (SE), and 95% credible intervals (CI) for the fixed and random effects of the fitted model.

	Mean	SE	95% CI	Mode
***Fixed effect***				
Intercept	−0.591	0.034	(- 0.660, – 0.521)	−0.592
***Random effects***				
Overdispersion (${\hat{\sigma }}_{r}^{2}$)	7.81	0.48	(6.91, 8.79)	7.76
Structured spatial effect (${\hat{\sigma }}_{V}^{2}$)	2,744.66	3,222.98	(232.72, 11,172.39)	693.99
Unstructured spatial effect (${\hat{\sigma }}_{U}^{2}$)	2,812.57	3,252.85	(255.58, 11,324.50)	634.07
Structured temporal effect (${\hat{\sigma }}_{\gamma }^{2}$)	29.54	27.93	(3.83,103.50)	10.36
Unstructured temporal effect (${\hat{\sigma }}_{\varphi }^{2}$)	132.00	168.00	(13.85,562.03)	35.02
Space-Time effect (${\hat{\sigma }}_{\delta }^{2}$)	4.57	0.77	(3.25,6.27)	4.36
***Fraction of variance***				
Overdispersion	0.0425			
Structured spatial effect ($V_{i}$)	0.0003			
Unstructured spatial effect ($U_{i}$)	0.0008			
Structured temporal effect ($\gamma_{t}$)	0.0219			
Unstructured temporal effect ($\varphi_{t}$)	0.0060			
Space -Time effect ($\delta_{it}$)	0.9286			

The mode of the posterior density for the precision of the Type-IV space-time interaction, $\delta_{it}$, is small capturing the persistent temporal dependence of the crime rates and the equally persistent effects of the neighbor´s rates on the level of homicide risk for a given district. The model accounts for non-linear effects both over time and across districts. The similarity of the modes of the independent, $U_{i}$, and structured district effects, $V_{i}$, indicates that in the absence of a space-time interaction term, none of these effects would appear as dominant, and that a between-district analysis of crime rates would not allow for the separation of heterogeneity and neighboring effects. Finally, the fact that the posterior density of the precision of the prior variance for the structured temporal effect, $\gamma_{t}$, has a smaller mode than the precision for the independent time effect, $\varphi_{t}$, reinforces the notion of persistent temporal trends in the homicide rates.

### Model checking

**Fig 5:**
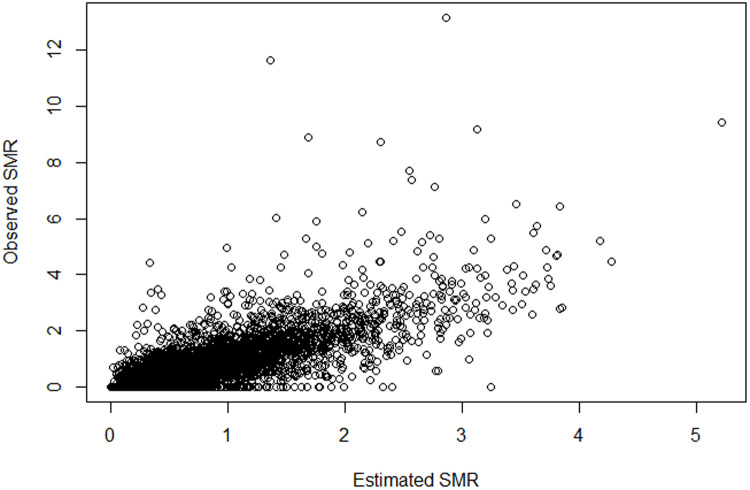
Comparison of fitted (x-axis) and observed SMRs (y-axis).

Model check was based on the posterior predictive distribution. Fig 4 plots the observed standardized mortality rates with the posterior fitted standardized mortality rates. The graph shows a relatively good correspondence of the model-based estimates with the observed standardized mortality rates (Fig 5).

### District-year evolution of relative risk

**Fig 6:**
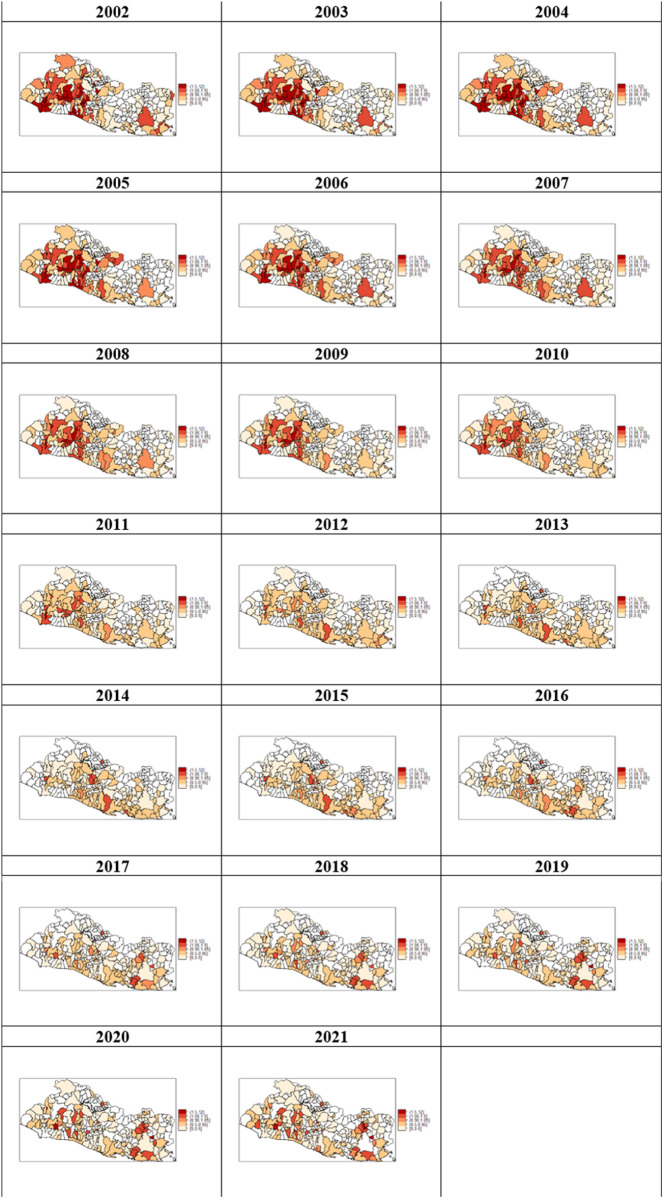
Posterior homicide rates (2002-2021). The legends in the maps correspond to the following values for the relative risk: Less than 0.50 (antique white), 0.50 to less than 0.95 (burlywood), 0.95 to less than 1.05 (salmon), 1.05 to less than 1.50 (brown), and 1.50 and more (red).

Fig 6 shows the map for the posterior district-homicide-rate by year.

The data in the map indicate that between 2002 and 2010, the geographic distribution of the homicide rate remained stable. With one exception, clusters of high-risk districts, including the capital city San Salvador (refer to map in Fig.1) together with other districts conforming the so-called San Salvador Metropolitan Area (AMSS), were located in the center-west side of the country. The AMSS concentrates more than half the country population and nearly 75% of total economic activity. The districts in the west side seemed to be part of a corridor connecting San Salvador with the country´s main sea-port located southwest on the Pacific coast, and with the Honduras border on the north. Fig 6 also shows clusters of medium to low risk districts located in the eastern side of the country including the third most populated district, San Miguel, connecting with several districts located on the Pacific southeast coast. The maps show a high-risk cluster of two neighboring districts in the country’s center-south. One of them is site for a maximum-security prison opened in 2003 and where the most influential gang leaders and members of powerful organized-crime groups were serving terms of imprisonment [63].

A change occurred to the spatial distribution of homicide risk in 2011 as seen in Fig 6. There was a reduction in the numbers of districts making up the cluster of highest relative risk connecting the capital city San Salvador with the Pacific coast on the southwest and with the Honduras border on the north. Most of the districts included in the highest-incidence clusters between 2002 and 2010, had the value of their relative risks reduced to 1. The district sitting the main security prison and its neighbor remained as a high-incidence cluster.

The 2012–2016 period covered the gang-government negotiations leading to the gang-truce in 2012 (refer to the introduction) which besides a sensible decline in the numbers of homicides enabled gangs to gain strong political influence. It also covered the Sánchez-Cerén’s government back off the truce in 2014 and its declaration of war against gangs as well. Fig 6 shows that compared to previous periods, the geographical distribution of homicide changed significantly during 2012–2016. Consistent with the decline in homicide rates following the gang-truce in 2012, there was a dispersal of high-crime areas resulting in three clusters of high homicide-risk. One comprised two districts located on the southwest side; a second cluster conformed by two center side districts bordering a lake; and the other was the district neighbor to the one where the maximum-security prison was located.

A shift of high-homicide clusters towards the eastern side of the country took place during the period 2017–2019. Fig 6 depicts two high-risk clusters surrounding San Miguel, the main regional eastern district, one by the north and two others by the south, on the Pacific coast. In 2017, the Sánchez-Cerén’s government began implementation of the “Plan El Salvador Seguro” within the framework of the “extraordinary security measures” (refer to the introduction). Note that the risk for the district that was neighbor to the one sitting the maximum-security prison dropped to 1. In 2017, a new maximum-security facility was opened in the southwest west district of Izalco to incarcerate members of the MS-13 gang. The leaders of this prison were responsible for the control of the routine-actions of the at-large gang members [64]. In 2019, the government transferred Barrio-18-gang leaders to this facility [65]. Izalco remained as a high-risk area in 2017–2019. Other high-risk clusters located in the west-center side of the country known for being gang strongholds appeared during this period.

The 2020–2021 period was characterized by a number of actions by the current Bukele’s government, including negotiations with gang leaders, aimed at setting the conditions for the implementation of the so called “Plan Control Territorial” and the further approval of the state of exception. As expected, the geographic distribution of homicide-risk did not vary relative to the one prevailing during the previous period.

**Fig 7:**
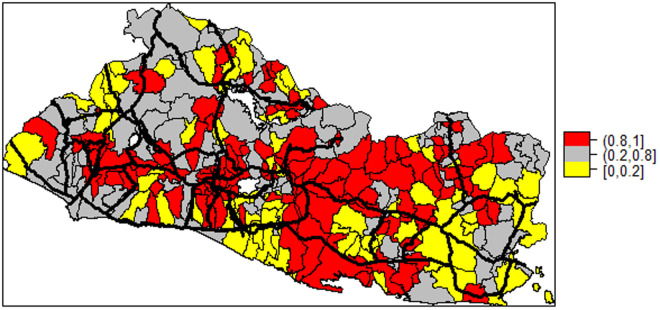
Posterior Probability of the Space-Time Effect.

Finally, Fig 7 shows the posterior probability of the space-time interaction effect being greater than cero, together with the country’s main road network, colored in strong-black. The data indicate those districts where temporal behavior of the homicide rate was affected by the temporal pattern of the homicide rates for their neighboring districts.

The districts with posterior probabilities greater than 0.8 for the space-time random effects (red-shaded) were located over a well-defined corridor running from the country’s southwest through to the north-east side with several bifurcations in between. There seems to be a close correspondence between the strength of the space-time random effect with the district location along a main road, be it either the Interamerican Highway, or the Coastal (“Litoral”) Highway along the Pacific coast, or the Northern Longitudinal Highway along the Honduras border, which cross the country in the west-east direction, or the Northern Trunk Highway that connects the capital city San Salvador with the Honduras border.

There are two major bifurcations of high homicide risk that merit attention. The first, is a cluster of districts located in the center west side of the country, between the Interamerican and the Coastal Highways, which includes the capital city San Salvador. The second connects districts located on the center east Pacific coast with northeastern districts in the direction of the Honduras border. These areas are known to be part of corridors for counterfeiting, drug trafficking and the smuggling of weapons.

The districts with posterior probabilities for the space-time random effects smaller than 0.2 (yellow-shaded) had more stable time trends, meaning that the variation in homicide rates within these places may have been dominated by spatial correlation relative to their neighboring districts.

**Fig 8:**
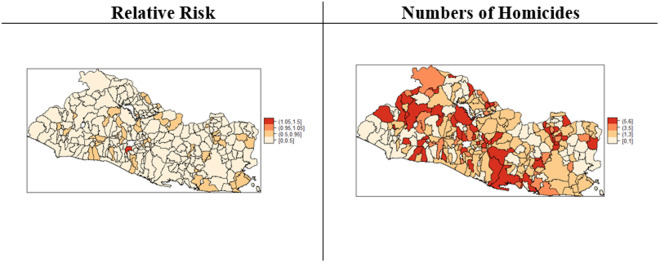
Posterior Predictive Distributions of Relative Risk and Numbers of Homicides – 2022.

Though the purpose of the fitted model in (1) and (2) was not forecasting any measure of homicide risk, I assessed the predictive posterior distribution of the standardized mortality rate by using the average expected numbers of homicides by district during the four-year period from 2018 to 2021 as an estimate of the expected numbers of homicides that might have been observed during the out-of-sample year of 2022. Similar to an ecological model where observed values for crime related variables are used as inputs for prediction, under a Bayesian approach it is always possible to make a prediction from the predictive distribution given the already observed data and the posterior distribution of homicide risk conditional on the posterior means of the random effects, and then derive a new predicted value for the number of homicides for a future period. Random effects are surrogates for any variable related to the district homicide risks. Fig 8 shows the posterior predictive distributions of the relative risk (left panel) and the numbers of homicides (right panel) for 2022.

Consistent with expectations, the left-hand panel of Fig 8 shows a generalized decrease in the relative risk of homicide across all districts. With the exception of a district with a small population, in 2022, all districts would have had relative risks with values below 1. The right-hand panel of Fig 8 shows the posterior predictive distribution of the numbers of homicides for 2022. The total predicted number of homicides was 947, almost double the official count of 495 homicides in 2022 [66], a finding that may provide support for either a generalized deterrence effect on violence, or an under reporting of homicides due to the security measures by the current government.

### Gangs and the space-time evolution of relative risk

**Fig 9:**
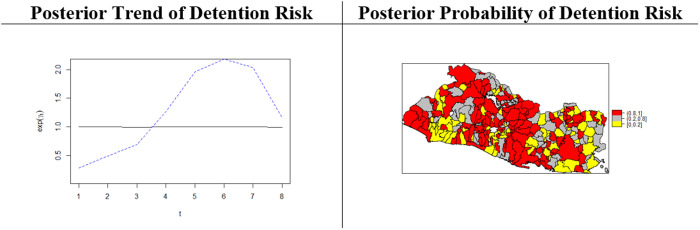
Temporal trend and posterior probability of the space-time effect, risk of gang- detention (2011-2018).

The clustering of high-risk districts suggests that the processes of formation, settlement, growth and expansion of gangs may have been a key factor in driving the space-time evolution of homicide. This presumed gang effect was partially assessed by fitting a Type IV interaction model to the data on gang detentions over 2011–2018. Fig 9 shows the map for the posterior district-relative-risks of detention for gang-membership by year (right hand side panel), and the posterior temporal trend (left hand side panel). Gang-detention rates were higher in 2017, the year when the war-on-gangs, enforced with the “extraordinary measures”, was fully implemented by the second FMLN government (refer to the introduction). Fig 9 also indicates clustering of districts by their gang-detention rates (right panel).

It seems reasonable to assume the increased risk for detention rates concentrating within districts where gangs had greater presence and that the contribution of these criminal groups to homicide rates was greater. Comparison of Fig 8 and Fig 9, suggests that this might be the case.

### Temporal evolution of relative risk

**Fig 10:**
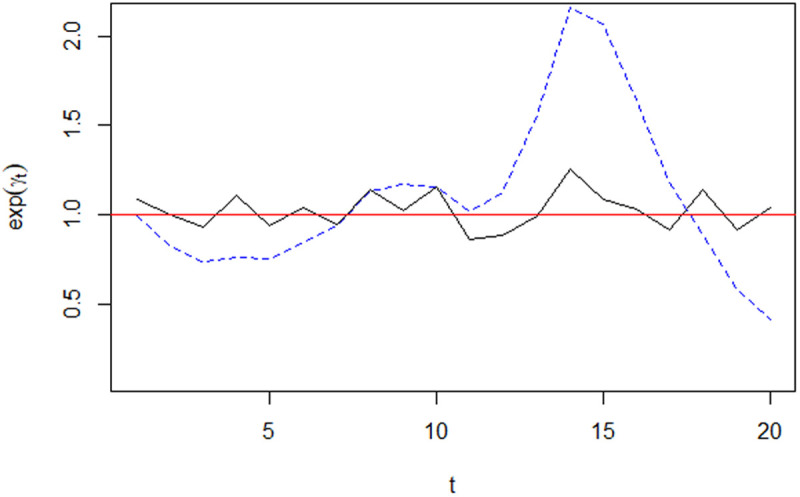
Posterior Temporal Trend for Relative Homicide Rates, 2002-2021.

Fig 10 shows the posterior temporal trend for homicide rates in El Salvador.

**Fig 11:**
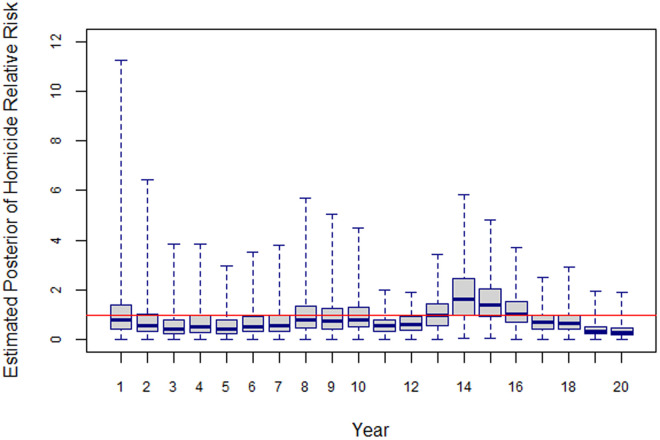
Posterior Relative Homicide Rates, 2002-2021.

The unstructured time effect, $\text{ exp}(\varphi_{t})$ (solid black line), shows some fluctuation around 1. On the other hand, the structured temporal (AR2) effect, $\text{exp}(\gamma_{k})$ (dashed blue line), shows a decreasing trend between 2002 and 2004 followed by an increasing one from 2005 through to 2015, and a declining trend since then. The boxplot in Fig 11 shows that the relative risk of homicide remained below 1 between 2003 and 2008, it was above 1 from 2009 to 2011, then went back to values below 1 during 2012–2013 (gang-truce period), increased to above unity values between 2014 and 2017, and has taken on values below 1 from 2018 onwards.

Together, the data in Fig 10 and Fig 11, suggest the presence of two cycles in the evolution of homicide during the 2002–2021 period. The first, characterized by relatively low rates, went from 2003 through to 2012 whereas the second, starting in 2013 and still going on at the time of writing. The cycles seem to last between 8 and 9 years. This finding reinforces the view that the efficacy and timing of government actions aimed at neutralize gangs, and the timing of government-gang negotiations have been amongst the drivers of the long-term behavior of homicide rates in El Salvador.

### Limitations

Government- restrictions on access to homicide data since March 2022, related to the exception regime prevented me from using district homicide data for 2022 and 2023 in my analysis. Also, the lack of data on gang detentions for the whole 2002–2021 period did not enabled me to fully assess the likely gang-homicide relationship.

A limitation of the random effect model used for analysis has to do with the fact that data on all the factors that create variation in homicide risk are not available across the 262 districts over the years within the period from 2002 to 2021. Reliable data on some of the traditional variables used to explain variation in violence related to social disorganization, relative socioeconomic disadvantage, and neighborhood environmental conditions might have been obtained from the 2007 national census records. Besides the census data being too old, it is uncertain whether the values of such variables would have remained the same over the study period. A number of technical and practical issues prevented us from using data from the national household surveys conducted by the central statistical office to obtain estimates for some of these variables, the most important being that it does not collect data for the 262 districts together with the unreliability of survey estimates for small districts. This was the reason underlying the use of a model with random effects to capture the impact of unobserved variables at the level of districts, time periods, or both.

The use of a Bayesian random effect approach is a strength of this paper. There are obvious statistical and computational challenges with pursuing a Bayesian modelling approach going from the specification of prior distributions to the inferences based in the estimates of posterior distributions for the model parameters and posterior predictive distributions for homicide risk. Random effects help address the issues arising from omitted variables by accounting for variability within the data.

Our choice of the district as the geographical unit for analysis has the potential to introduce bias due to the modifiable area unit problem (MAUP) as our results might differ from those obtainable from any other analysis based on different geographical units such as regions, provinces (departments), neighborhoods, or census tracks. This was demonstrated in [66–68] for Brazil and Argentina, respectively. In El Salvador, the district is the lowest geographical level for dissemination of police data. Another possibility is that point level data be available at the point level. Using original point data is a way to mitigate the impact of MAUP. To the best of my knowledge, official geopositioned data for each of the 65,328 homicides that have occurred in El Salvador during the twenty years and across the 262 districts included in this study is nonexistent. A major issue with this research has to do with assuming that homicide risk is uniform within each district. This is not the case, especially for large districts where some sort of selectivity process might drive decisions by gangs as to where to settle in or where to carry out their activities. Had point-level data been available, one might have been able to identify high risk areas within districts which in turn would have helped in better understanding the nature and strength of between-district autocorrelations.

## Conclusions

This research was to study the evolution of homicide in El Salvador within a hierarchical Bayesian space-time framework over the 2002–2021 period. The Bayesian approach allowed me to account formally for uncertainty at each of the data, process and parameter levels, by paying attention to localized patterns that arise from incorporating space-time interactions. In particular, I dealt with the case when district-level deviations from the global trends are assumed to be correlated with their neighbors, both in space and time, and modeled hidden factors whose effects go beyond the geographical border of one or more districts and also persisted for more than one year, as well.

The combined space-time approach led me to conclude that variation in homicide rates was consistent with the idea of homicide as an outcome from the unobserved activities of gangs, and the interactions they have established with politicians and governments over time. Also, my findings confirm that unobserved variables on the socio-economic composition of the districts intervened in the realizations of the interactions.

Altogether, the findings provide support to a model with a term for space-time interaction. The model did fit the data well and supported the notion that unobserved covariates related to the district patterns of homicide have evolved over time. These covariates might relate with the efficacy and timing of government actions aimed at neutralizing gangs in their capacity to produce violence and the likely responses of gangs towards the government initiatives. Two cycles seemed to be present in the evolution of homicide over the 2002–2021 period. The first was characterized by relatively low rates and went from 2003 through to 2012. The second, started in 2013 and still is going on at the time of writing. This finding reinforced the view that the efficacy and timing of government actions to neutralize gangs, and the timing of government-gang negotiations have been key drivers of the long-term behavior of homicide rates in El Salvador.

The clustering of high-risk districts suggests that the processes of formation, settlement, growth and spread of gangs have been key in driving the space-time evolution of homicide. This presumed gang effect was detected from data on gang detentions over 2011–2018. Gang detentions in districts with concentrated high homicide risk provided evidence on the sizeable contribution of these criminal groups to the formation of space-time clusters of violence.

We provide empirical evidence for the observed decline in homicide rates since 2022 being related to the set of measures implemented by the current government to regain the territorial and social control of the areas where gangs committed murders, disappeared people, and extracted rents from both, residents and local businesses. However, our findings suggest the possibility of underreporting of around half of the total numbers of homicides that occurred in 2022.

The main implications deriving from this research relate to the way the current government has implemented the anti-gang measures. By incarcerating around 80,000 alleged gang members and collaborators the government not only has incapacitated gangs as the main producer of violence, but also has had a generalized deterrence effect on other forms of crimes. An immediate effect has been a reduction of the fear of crime among the general population. As crime-related gang activity, allegedly the major crime problem of the Salvadoran society, seems to have been brought under control, the government must focus on developing initiatives to build collective efficacy, in particular among the most socially and economically disadvantaged communities to avoid the formation of gang-type groups in the medium to long term. Other implications of a more political nature are beyond the scope of this research.

## Supporting information

S1 File Homicide_Rates_per_10,000, 2002–2021. (CSV)

S2 File Detentions_Alleged_Gang_Members_2011–2018. (CSV)

S3_File Official_population_projections_2002–2022. (CSV)

S4 File S4_File_Observed and expected homicides, 2002–2021. (DBF)

S5 File Computer code – Local Moran statistic by year. (TXT)

S6 File Computer code – Spatio-temporal models and maps. (TXT)

S7 File Temporal adjacencies. (TXT)

S8a File El Salvador municipal map-dbf. (XLSX)

S8b File El Salvador municipal map-shp. (XLSX)

S8c File El Salvador municipal map-shx. (XLSX)

S9a File El Salvador homicide rates with geographic-dbf. (XLSX)

S9b File El Salvador homicide rates with geographic-shp. (XLSX)

S9c File El Salvador homicide rates with geographic-shx. (XLSX)
